# Chemically fueled materials with a self-immolative mechanism: transient materials with a fast on/off response†

**DOI:** 10.1039/d1sc02561a

**Published:** 2021-06-21

**Authors:** Patrick S. Schwarz, Laura Tebcharani, Julian E. Heger, Peter Müller-Buschbaum, Job Boekhoven

**Affiliations:** Department of Chemistry, Technical University of Munich Lichtenbergstraße 4 85748 Garching Germany job.boekhoven@tum.de; Lehrstuhl für Funktionelle Materialien, Physik Department, Technische Universität München James-Franck-Str. 1 85748 Garching Germany; Heinz Maier-Leibnitz Zentrum (MLZ), Technische Universität München Lichtenbergstr. 1 85748 Garching Germany; Institute for Advanced Study, Technical University of Munich Lichtenbergstraße 2a 85748 Garching Germany

## Abstract

There is an increasing demand for transient materials with a predefined lifetime like self-erasing temporary electronic circuits or transient biomedical implants. Chemically fueled materials are an example of such materials; they emerge in response to chemical fuel, and autonomously decay as they deplete it. However, these materials suffer from a slow, typically first order decay profile. That means that over the course of the material's lifetime, its properties continuously change until it is fully decayed. Materials that have a sharp on–off response are self-immolative ones. These degrade rapidly after an external trigger through a self-amplifying decay mechanism. However, self-immolative materials are not autonomous; they require a trigger. We introduce here materials with the best of both, *i.e.*, materials based on chemically fueled emulsions that are also self-immolative. The material has a lifetime that can be predefined, after which it autonomously and rapidly degrades. We showcase the new material class with self-expiring labels and drug-delivery platforms with a controllable burst-release.

## Abstract 2

We describe a design strategy for transient materials with a predefined lifetime and a fast on–off-response.

## Graphical abstract

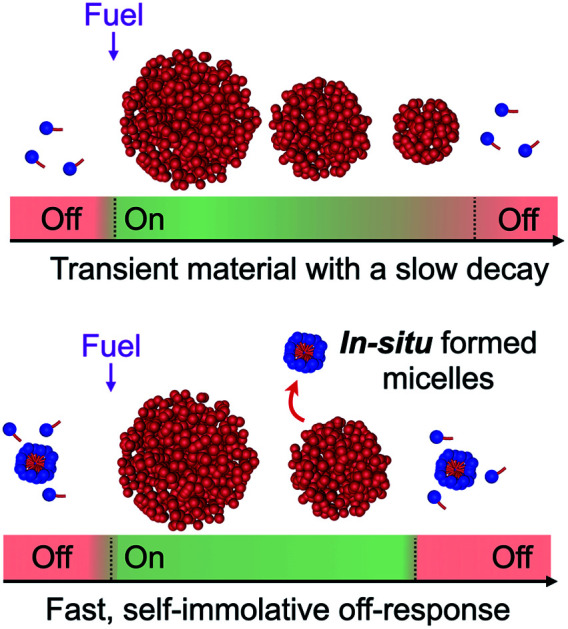

## Introduction

**Scheme 1:**
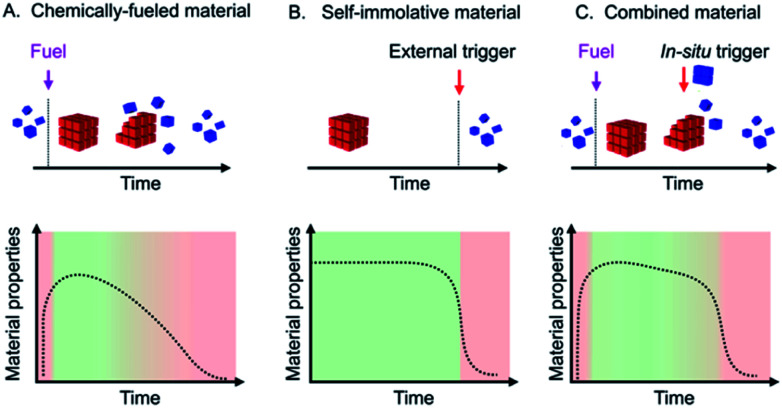
Schematic representations of a chemically fueled material (A), a self-immolative material (B), and a combined material (C) with their respective evolutions of material properties.

Transient materials retain their function over a defined period and dissolve or resorb when their task is fulfilled.^1^ They are particularly powerful in medicine, *i.e.*, as a scaffold that aids the body to regenerate lost tissue or as a delivery system for therapeutics.^2^ They have also gained popularity in electronics as temporary circuits that disintegrate after a predefined time.^1,3^ These materials degrade by a range of structure-dependent biodegradation processes.^4^ A different approach for the generation and degradation of transient materials is through chemically fueled materials. These materials are regulated by a fuel-driven chemical reaction cycle, *i.e.*, in the cycle, building blocks for the materials are activated at the expense of chemical fuel, and the building blocks spontaneously deactivate. When a finite amount of fuel is added to such a system, a material emerges, and it autonomously decays when it runs out of fuel.^5^ The building blocks of these materials are typically self-assembling molecules and yield supramolecular materials like fibers,^6^ vesicles,^7^ micelles,^8^ colloids,^9^ oil-or coacervate based droplets,^10^ nanoparticles,^11^ hybridized DNA^12^ and others. We and others applied chemically fueled assemblies as transient materials, *e.g.*, as self-erasing inks,^6*c*,13^ drug delivery platforms,^10*d*^ solutions containing macrocycles,^14^ transient hydrogels,^6*a*–*c*,12*a*,15^ supramolecular polymers,^16^ transient emulsions,^10*a*–*d*^ transient photonics^17^ and temporary nanoreactors.^7*c*,8*a*,18^ These fuel-driven supramolecular materials, but also other approaches towards transient materials, typically decay *via* first- or zeroth-order kinetics. The material and its properties will thus decay over its entire lifetime (Scheme 1A). Such a constant decay profile can be disadvantageous for applications that require a fast on-off response, *i.e.*, materials in which the period of switching off is only a fraction of the total lifetime of the material. For example, a transient electronic circuit that is operational for its entire lifetime and then rapidly dissolves is more useful than one that decays gradually after its emergence. Similarly, it would be desirable that self-degrading packaging retains its material properties and rapidly decays when its lifetime is over.

Self-immolation partly addresses this challenge by making use of a self-amplifying decay mechanism which found widespread application in hydrogels,^19^ drug delivery,^20^ antibiotics,^21^ fluorescent labels,^22^ temporary linkers,^23^ microcapsules and membranes,^24^ degradable plastics,^25^ sensors^26^ and responsive polymers.^27^ However, self-immolative materials do not decay autonomously but instead require an external trigger that initiates the self-amplifying cascade (Scheme 1B).

In this work, we thus explore materials that make use of the best of both: we describe the synthesis of chemically fueled materials with self-amplifying decay mechanisms to accelerate their off-response (Scheme 1C). We make use of two simultaneously operating feedback mechanisms of the material on its regulatory kinetics: one feedback mechanism ensures that the material decays slowly with linear kinetics, while a second feedback mechanism ensures a rapid autocatalytic decay once a threshold level is reached. In contrast to self-immolative materials, the trigger for the degradation is released *in situ* by the reaction cycle. In other words, the addition of a trigger is not necessary as the autocatalytic decay occurs as soon as the *in situ* release of the trigger reaches the aforementioned threshold. The result is a set of materials, turbid emulsions, self-erasing labels, and drug-releasing hydrogels that autonomously decay after their lifetime with a very fast, autocatalytic response. Specifically, the decay time is less than 10% of the total material's lifetime.

## Results and discussion

**Fig. 1:**
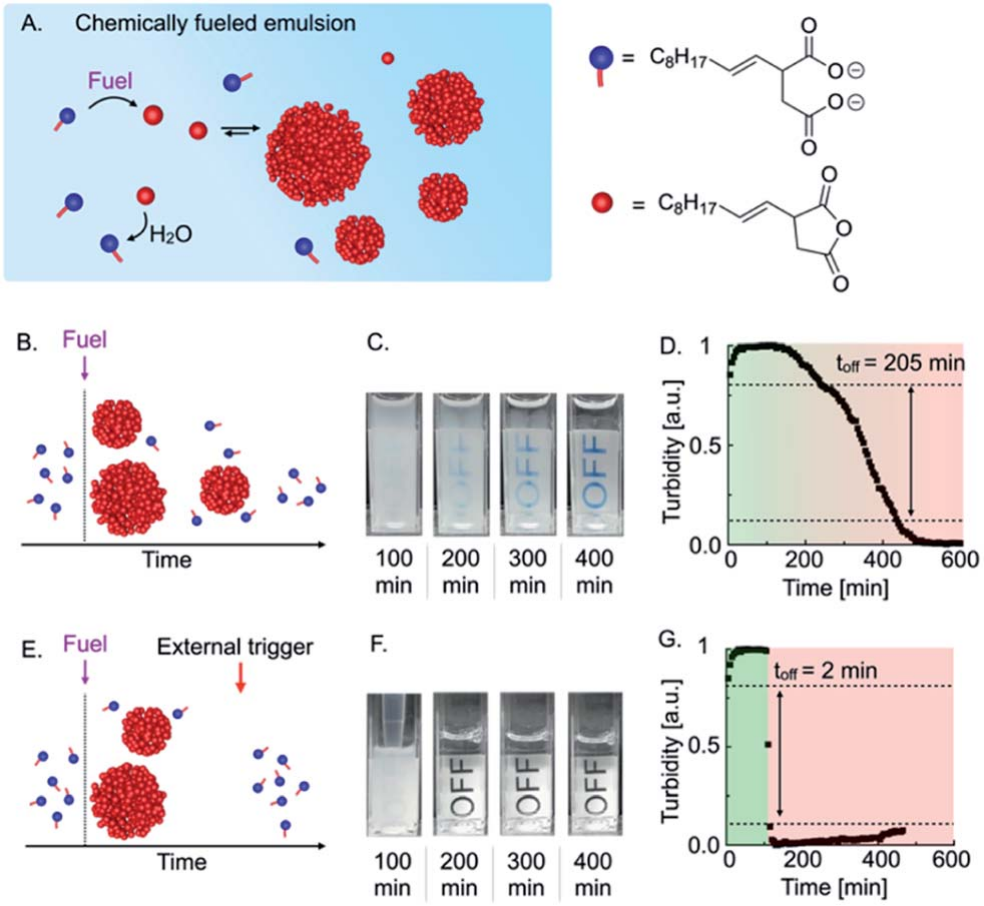
Triggered self-immolation of a chemically fueled, active emulsion. (A) Schematic representation of a chemically fueled reaction cycle based on 2-decen-1-ylsuccinic acid as precursor and EDC as chemical fuel. (B) Schematic representation of the temporal course of a chemically fueled emulsion. (C) Webcam images of 7.5 mM precursor fueled with 2 mM EDC. (D) Grey-value analysis of 7.5 mM precursor fueled with 2 mM EDC. (E) Schematic representation of the temporal course a self-immolative emulsion that rapidly degrades in response to the addition of the surfactant precursor as an external trigger. (F) Webcam images of 7.5 mM precursor fueled with 2 mM EDC and triggered with 20 mM precursor after 100 min. (G) Grey-value analysis of 7.5 mM precursor fueled with 2 mM EDC and triggered with 20 mM precursor after 100 min.

The basis of our autonomous self-immolative material is a transient emulsion formed by a chemically fueled reaction cycle (Fig. 1A). The reaction cycle uses a condensing agent (1-ethyl-3-(3-dimethylaminopropyl)carbodiimide hydrochloride (EDC or fuel) as a chemical fuel to convert the 2-decen-1-yl-succinate (precursor) into its corresponding anhydride (activation reaction). The anhydride is unstable in the aqueous media and hydrolyzes back to the precursor (deactivation reaction). Thus, when a finite amount of fuel is added, the anhydride emerges and decays again as fuel is depleted. Above its solubility, the anhydride phase separates into micron-sized oil droplets, which results in the formation of a turbid emulsion (see ESI, Fig. 1†).^10*a*,10*b*,10*d*^ As the anhydride deactivates, the total droplet material does too, resulting in a decrease in turbidity until a transparent solution is obtained (Fig. 1B). Thus, the loss of turbidity can serve as a measure for the state of the material. The anhydride hydrolysis proceeds with a characteristic zeroth-order decay because the droplets protect the anhydride molecules from hydrolysis.^9,10*d*^ Consequently, hydrolysis only occurs on the fraction in solution, which is constant and equal to the anhydride's solubility.

When we added 2 mM EDC to 7.5 mM precursor, the solution immediately turned turbid (Fig. 1C). Over the course of roughly 400 min, the turbidity of the emulsion decreased continuously, which we quantified by measuring the grey value of the time-lapsed photographs in selected areas and found a characteristic constant decay of the emulsion's turbidity (Fig. 1D). We considered the material as “turbid” above a grey value of 0.8 and “optically clear” below 0.1. Using this definition, we could calculate that the material spends roughly 205 min transitioning from turbid to optically clear. We refer to this value as the “off-response,” *i.e.*, the time it takes to switch off the material.

**Fig. 2:**
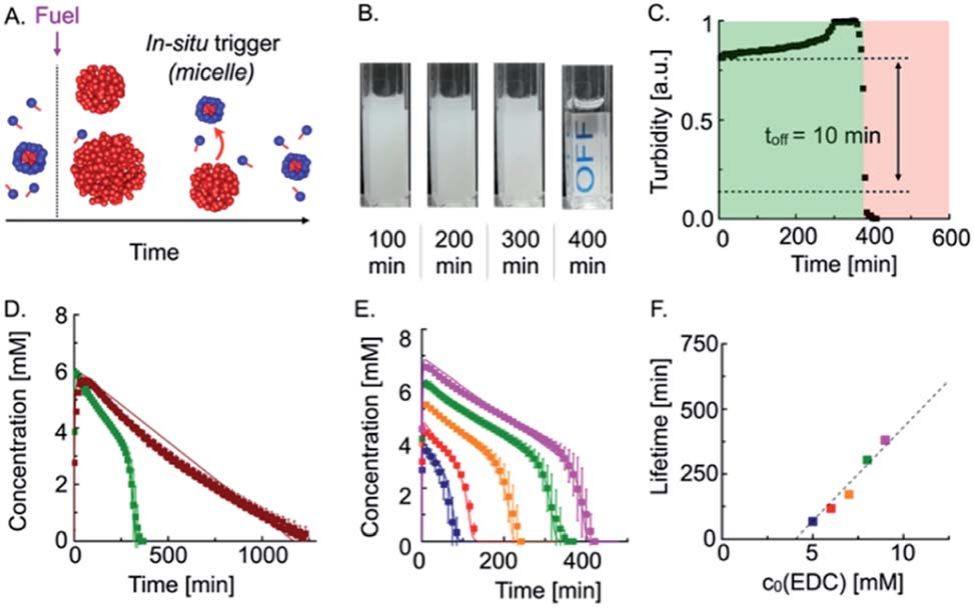
Autonomous self-immolation based on an *in situ* trigger release. (A) Schematic representation of an autonomous self-immolative material. (B) Webcam images of 20 mM precursor fueled with 9 mM EDC. (C) Grey-value analysis of 20 mM precursor fueled with 9 mM EDC. (D) Anhydride concentration profiles of 7.5 mM (burgundy red) and 20 mM precursor (green) fueled with 8 mM EDC. (E) Anhydride concentration profile of 20 mM precursor fueled with different amounts of EDC. Markers represent HPLC data; solid lines represent data calculated by the kinetic model. (F) Lifetime of the turbidity against initial fuel concentration (markers). The dashed line is added to guide the eye.

**Fig. 3:**
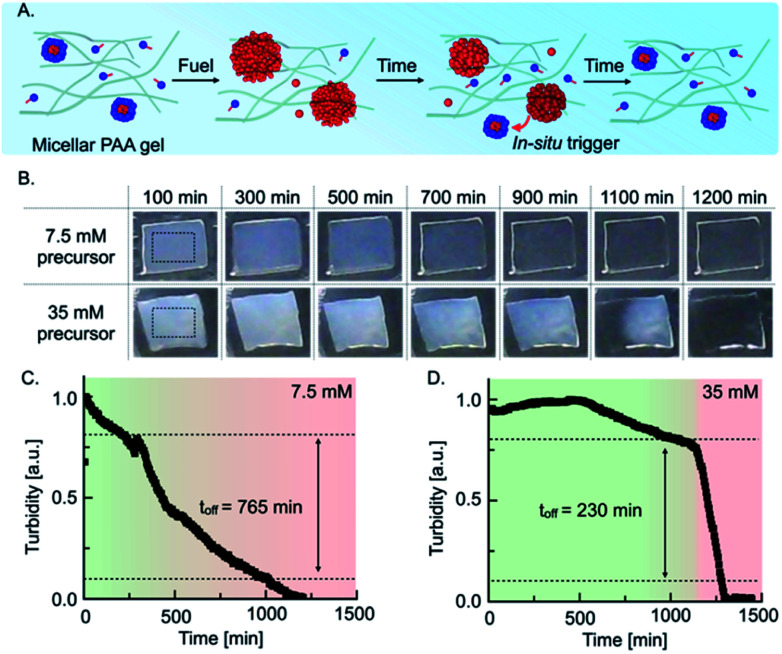
Self-immolative emulsion embedded in a polyacrylamide gel and its application as a self-expiring label. (A) Schematic representation of a self-immolative emulsion immobilized in a polyacrylamide (PAA) hydrogel. (B) Time-evolution of 15% PAA hydrogels containing 7.5 mM and 35 mM precursor spray-coated with 200 μL of a 3 M EDC stock solution. Rectangles in the first images represent the region of interest (ROI) considered for the grey value analysis. Grey value analysis of (C) 7.5 mM and (D) 35 mM precursor in 15% PAA hydrogels.

**Fig. 4:**
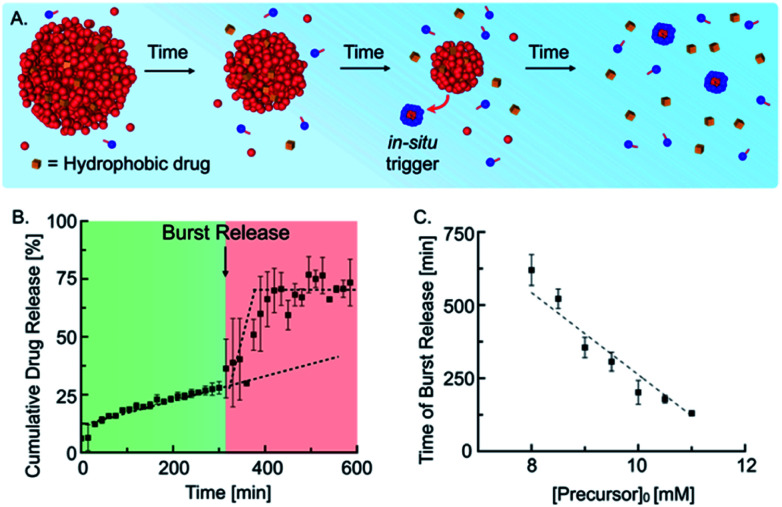
Self-immolative emulsion as drug delivery platform. (A) Schematic representation of the temporal course of a self-immolative drug delivery platform. (B) Cumulative drug release of 15 mM anhydride droplets and 9 mM precursor embedded in an agar–agar gel. (C) Time of burst release in dependence on initial precursor concentration in the agar–agar gel. Measurements were performed in triplicates and lines were added to guide the eye.

In order to obtain a sharper off-response, like self-immolative materials, we tested whether the addition of an external trigger could induce the degradation of the material (Fig. 1E). We hypothesized that the addition of a surfactant could increase the solubility of the anhydride and thereby accelerate its deactivation through hydrolysis.^28^ For common and inverse micelles, a micelle-catalyzed formation of micelle-forming components results in an autopoietic micellar system, *i.e.*, the micelles catalyze the formation of their own building blocks.^29^ Analogously, we hypothesized that the precursor at high concentrations could act as a surfactant that forms micelles and helps to dissolve the anhydride which accelerates the hydrolysis and results in the release of more surfactant. Indeed, by means of Nile Red assay and DLS measurements, we found that the precursor could form micelles of roughly 7 nm in diameter in the absence of oil droplets above a critical micelle concentration of roughly 1 mM (CMC, see ESI, Fig. 2 and 3†). A CMC typically increases in the presence of oil droplets as the surfactant first saturates the surface of the droplets before forming micelles.^30^ Thus, we also measured indirectly the CMC in the presence of oil droplets. Indeed, we found a CMC of roughly 11 mM precursor when samples were fueled with 2 mM EDC (see ESI, Fig. 4 and notes†). We hypothesized that the addition of precursor as an external trigger could result in an increase in the anhydride solubility. That increase would accelerate the hydrolysis that further releases precursor, which is the basis of a self-immolative cascade. To prove whether the precursor can be used as an external trigger, we fueled 7.5 mM precursor with 2 mM EDC and added a concentrated solution of precursor after 100 min (Fig. 1F). In contrast to the experiment without the addition of a trigger, we observed that the emulsion almost immediately turned transparent. We quantified the self-immolation by measuring the grey values of a selected area in the images and calculated an off-response of 2 min (Fig. 1G). From these findings, we conclude that the excess of precursor can form micelles that solublize the anhydride oil resulting in its rapid hydrolysis. Thus, the addition of excess of precursor can serve as a trigger for self-immolation.

As the trigger for self-immolation of the emulsion is also produced by the deactivation reaction of the reaction cycle, we tested if we could design it such that the trigger is generated *in situ* (Fig. 2A). In our design, we start with a solution of precursor above its CMC, *i.e.*, a micellar precursor solution. To this solution, we add fuel to induce droplet formation. As the micelle-forming precursor is partly convertered into droplet-forming product, the micellar solution turns into a turbid emulsion without micelles. As the deactivation of the anhydride proceeds, the concentration of the precursor in the solution with droplets increases until it reaches the CMC of roughly 11 mM. At that point, the micelles will reappear and act as an *in situ* generated trigger for the solubilization of the emulsion.

Indeed, when we fueled 20 mM precursor with 9 mM EDC, the turbidity remained mostly constant, but, after 400 min, suddenly and rapidly decreased (Fig. 2B). Quantification of the grey value showed that the off-response took 10 min, which equals only 2.5% of the total lifetime (Fig. 2C). In other words, the emulsion retains its original properties 97.5% of the time, and then it rapidly degrades.

To quantitatively understand the mechanisms at play, we performed high-performance liquid chromatography (HPLC) of our transient emulsions (7.5 mM precursor with 8 mM EDC) and quantified the anhydride and EDC concentration profiles (Fig. 2D and ESI, Fig. 5†). Under these conditions, the precursor is not able to form micelles when droplets are present. We found that the anhydride concentration increased rapidly in the first 30 min at the expense of EDC, which is consumed within the same timeframe. From there on, the anhydride concentration decayed linearly until no more anhydride was detected after roughly 1400 min. We can explain the linear decay of the anhydride with a self-protection mechanism which was described in previous work.^9,10*c*^ In this mechanism, the anhydride phase separates into droplets and is thereby physically separated from the aqueous phase. Thus, hydrolysis can only take place on the fraction that remains in the aqueous solution, which results in a linear decay of the anhydride until all droplets are dissolved. This relation implies that the decay rate of the emulsion is dependent on the anhydride solubility, which is constant. Indeed, we were able to fit and accurately predict the kinetics of our reaction cycle with a kinetic model that takes into account this self-protection mechanism (see ESI, Fig. 6, notes, and ESI, Table 2†).

When we fueled a solution of 20 mM precursor with the same amount of fuel, *i.e.*, a solution of precursor that contained micelles, we found a similar rapid increase in anhydride concentration until all fuel has been depleted (Fig. 2D, green trace). Then, the anhydride concentration decays linearly, which implies that the self-protection mechanism is also present in this experiment. However, after 300 min, the negative slope of the anhydride concentration as a function of time started to increase and deviate from a linear decay. The acceleration of the hydrolysis rate kept on increasing until no more anhydride was detected, pointing towards an autocatalytic hydrolysis mechanism.

We explain the autocatalytic behavior by the following mechanism. Before the addition of fuel, the solution of the precursor contains micelles. The conversion of the precursor into its corresponding anhydride decreases the precursor concentration in solution to values below its CMC. Consequently, shortly after the addition of fuel, the solution contains droplets and most likely no micelles. As the reaction cycle proceeds, the anhydride concentration decays linearly, and thus the concentration precursor increases *vice versa*. After 300 min, the precursor reaches its CMC, and micelles start to form, which act as a phase-transfer catalyst and solubilize the anhydride which significantly increases the anhydride hydrolysis rate. Similar to reported models of micellar phase-transfer catalysis, it is likely that mixed micelles composed of precursor and anhydride are formed.^31^ However, the hydrolysis reaction of the anhydride does not occur at or in the micelles but in the bulk solution.^31,32^ As the anhydride hydrolysis rate increases, the concentration of micelles further increases, thereby further accelerating the hydrolysis rate. This mechanism explains (1) the sudden onset of acceleration of the hydrolysis and (2) the ever-increasing hydrolysis rate due to autocatalysis.

To quantitatively verify our proposed mechanisms, we wrote a kinetic model that describes the activation reaction, deactivation reaction, and the self-protection mechanism, which is activated when the anhydride passes its solubility of 0.025 mM in the absence of micelles (see ESI, Fig. 6, Table 2 and notes†). The model captures the evolution of the anhydride in the absence of micelles very well. Then, we adjusted the kinetic model to also describe the autocatalytic behavior. Autocatalytic micellar systems have been well described with kinetic models relying on classical micellar catalysis^33^ as well as on phase-transfer catalysis.^31,32,34^ We found good agreement with the experimental data using a phase-transfer catalysis model in which micelles enable the insoluble anhydride the transition in the aqueous phase. We observed that the autocatalytic decay initiated when the concentration precursor reached a threshold concentration of 16.5 mM, which we considered as the CMC of the system under these conditions. Thus, in our kinetic model, we described that above 16.5 mM, every additional precursor molecule released by hydrolysis increases the solubility of the anhydride. We found that we could fit the data well by defining an effective solubility (*S*_eff_) above the CMC. It is calculated based on the constant solubility of the anhydride below the CMC (*S*_0_) and increases as a function of the amount of precursor above the CMC. This increase scales with a factor of 0.1, which we refer to as the solubilization capacity (SC) of the surfactant precursor. The effective solubility can then be calculated following the equation *S*_eff_ = *S*_0_ + SC × ([precursor] − CMC) (see ESI, notes†). For example, if the precursor concentration was 17.5 mM, *i.e.*, 1 mM above its CMC, the effective anhydride solubility increased from 0.025 mM to 0.125 mM. The increase in solubility accelerates the hydrolysis, which again increases the precursor release rate. The solubilization capacity SC = 0.1 implies that roughly ten precursor molecules are used to solubilize a molecule of anhydride. Our kinetic model calculated that the maximum acceleration of the hydrolysis rate towards the end of the self-immolation regime was 10-fold higher compared to the chemically fueled material without self-immolation (see ESI, Fig. 7†).

Excited by the accurate prediction of the data by our kinetic model, we tested the system for different initial amounts of fuel, *i.e.*, 5–9 mM EDC (Fig. 2E). With an increasing amount of fuel, the maximum anhydride concentration increased. The subsequent slope of the hydrolysis was nearly equal for all experiments and we slightly adjusted our kinetic model by adjusting the anhydride solubility *S*_0_ (see ESI, Table S3†). Interestingly, the onset of the autocatalysis occurred at roughly the same precursor threshold (16.5 mM) and resulted in the complete hydrolysis of the anhydride within 20 min. The kinetic model allowed us to accurately fit the anhydride concentration profiles for different fuel concentrations, further validating the mechanism through micellar autocatalysis. Moreover, we demonstrated the autocatalytic nature of the micelles by the addition of different amounts of the precursor as a seed which resulted in an earlier acceleration of the hydrolysis rate and shorter lifetime (see ESI, Fig. 8†). We were able to tune the lifetime of the cycle from 60 min up to 400 min (Fig. 2E), and we found that the lifetime of the turbidity scaled linearly with the initial fuel concentration (Fig. 2F and ESI, Fig. 9†).

The quantitative understanding of the mechanism allowed us to test the autonomous self-immolative emulsion in two materials. First, we designed a transient label that self-erased after fueling it (Fig. 3A). For the chemically fueled label without self-immolation, we embedded 7.5 mM precursor in a 15% polyacrylamide hydrogel and used a spray gun to coat these gels with 200 μL of a 3 M EDC stock solution. Upon spray-coating, the hydrogel became turbid, indicating that the droplets emerged in the polymer hydrogel. Thus, we imaged the evolution of the turbidity using a webcam set-up (Fig. 3B). We observed that the turbidity of the gel constantly decreased, and the label became transparent after roughly 900 min (Fig. 3B). To implement the self-immolation mechanism in the label, we increased the precursor concentration to 35 mM. When we fueled this label, we observed that it remained turbid for roughly 1100 min, after which the turbidity suddenly disappeared from one edge to the other, and the label became transparent. We assumed that the deactivation from one side to the other was caused by an inhomogeneous distribution of the fuel on the surface of the gel, *i.e.*, the turbidity in a region with less fuel is expected to degrade earlier as micelles reappear earlier. Grey value analysis of the turbidity validated that the turbidity of the common chemically fueled label containing 7.5 mM precursor constantly decreased with an off-response of 765 min which, corresponds to roughly 70% of its lifetime (Fig. 3C). In contrast, the combined material with the self-immolation mechanism (35 mM precursor) was constantly turbid for roughly 1100 min followed by a sharp decay with an off-response of 230 min, which corresponds to a degradation time of roughly 20% of its lifetime (Fig. 3D). From these findings, we can conclude that the self-immolation of the emulsion by the formation of micelles remains function in a polymer hydrogel. We observed that the self-immolation is slightly slower, but the off-response still remains roughly 3.5-fold faster than in a common chemically fueled material. Such materials could find application as self-expiring labels, *e.g*., an expiration date for perishable food or as an entrance- or bus ticket.

Next, we tested whether our self-immolative materials could be used to alter the profile of the release of hydrophobic drugs. Emulsions are frequently used for the sustained release of drugs, and we reported recently that droplets of a hydrolyzable oil could release drugs with zeroth-order kinetics.^10*d*^ In the self-immolative system, we expected a similar linear release of the drug followed by a sudden burst release when self-immolation commences (Fig. 4A). We prepared the drug delivery platform by embedding 15 mM anhydride droplets loaded with 25 μM of the hydrophobic drug Nimesulide (a common nonsteroidal anti-inflammatory drug) in an agar–agar gel (see ESI, Fig. 10†). To implement the self-immolation mechanism in the drug delivery platform, we added 9 mM of the precursor to the hydrogel and supernatant. Next, we measured the cumulative drug release with analytical HPLC (Fig. 4B) which showed a drug release with zero-order (linear) kinetics for the first 300 min. After 300 min, the trend suddenly accelerated, and the remaining 45% of the drug was released over the course of roughly 50 min. The triplicate measurement of the cumulative drug release showed increased deviations in the burst release regime (see ESI, Fig. 11† for the individual traces). We explain this behavior by small concentration inaccuracies which significantly influence the time of the burst release due to the autocatalytic nature of the mechanism. After 350 min, the drug concentration remained stable at an approximate cumulative release of roughly 70%. We assumed that the time of the burst release could be tuned by a variation of the initial precursor concentration in the gel and supernatant. Indeed, we found that an increase in the initial precursor concentration, *e.g.*, from 9 mM to 11 mM, reduced the time of the burst release from roughly 300 min to 125 min (see ESI, Fig. 12†). Moreover, we found that the time of the burst release shows a linear dependence on the initial precursor concentration (Fig. 4C). This relation enables us to predict the timepoint of the burst release for any initial precursor concentration using our kinetic model. In summary, the designed drug delivery platform releases the loaded hydrophobic drug following a unique constant-then-burst mechanism which can be tuned by the amount of initial precursor.

## Conclusions

We demonstrated that self-immolation can be designed in chemically fueled materials. The combined concepts result in a new type of transient materials: autonomous self-immolative materials, *i.e.*, materials that are transient and switch themselves off autonomously through a rapid, self-amplifying trigger. We showcase our finding with a transient emulsion that is regulated through a reaction cycle. We designed it such that the cycle releases a trigger for the self-immolation *in situ*. Unlike other transient emulsions, the self-immolative mechanism results in a very rapid off-response that we can accurately control. We preliminarily demonstrated the use of our finding as a self-expiring ticket and a drug delivery platform. In future work, we will implement self-immolation mechanisms to rapidly degrade other types of assemblies such as fibers or coacervate-based droplets. We envision that precise control over the degradation of the material could be used to create complex material behavior such as oscillations and patterns.

## Materials and methods

### Materials

2-Decen-1-ylsuccinic anhydride (anhydride) was purchased from TCI chemicals. 1-Ethyl-3-(3-dimethylamino-propyl)carbodiimide (EDC), 2-(*N*-morpholino)ethane-sulfonic acid (MES buffer), trifluoroacetic acid (TFA), Nimesulide, ammonium persulfate (APS) and Nile Red were purchased from Sigma-Aldrich. We purchased agar–agar, *N*,*N*′-methylene-bis-acrylamide, tetramethylethylenediamine (TEMED) and acrylamide from Carl Roth. All chemicals were used without any further purification unless otherwise indicated. High performance liquid chromatography (HPLC) grade acetonitrile (ACN) was purchased from VWR.

### Synthesis of the succinate precursors

5 mL of 2-decen-1-ylsuccinic anhydride (anhydride) was suspended in 30 mL MQ water and stirred for 3 days. Subsequently, the reaction mixture was freeze-dried and the corresponding 2-decen-1-ylsuccinic acid (precursor) was stored at −20 °C until further use.

### Sample preparation

We prepared highly concentrated stock solutions of the acid precursor in 0.2 M MES buffer and adjusted the pH to pH 6.0. We used 35 mM, 20 mM and 7.5 mM stock solutions of the precursor. Stock solutions of 1.0 M and 3.0 M EDC were prepared freshly before each experiment by dissolving the powder in MQ water. We prepared 5 mM stock solutions of Nimesulide by dissolving the drug in acetonitrile. All stock solutions were stored at 8 °C until further use. The reaction cycles were started by the addition of the appropriate amount of EDC stock solution to the precursor stock solution. All experiments were performed at 25 °C.

### Webcam set-up and quantification of turbidity

We used a webcam-setup with Timelapse software to take images of the substrates in a 5 min interval. We analyzed the turbidity of the substrates using the grey value analysis tool of ImageJ. The raw data of the grey values was normalized to 1 to account for differences in the background and lighting. The kinetics in cuvettes (see Fig. 1C, F and 2B) were performed in an incubator at 25 °C to account for the temperature dependency of the hydrolysis rate. The constant temperature allowed us to precisely determine the lifetimes of turbidity (see Fig. 2F). The imaging of the gels was performed at room temperature (see Fig. 3B).

### Polyacrylamide (PAA) gel preparation

We prepared a 7.5 mM and 35 mM precursor solution containing 30% (29 : 1) acrylamide by dissolving 5.80 g acrylamide, 0.20 g *N*,*N*′-methylene-bis-acrylamide and the precursor (38,43 mg and 179,32 mg, respectively) in 20 mL of 200 mM MES buffer at pH 6. We diluted the stock solution with the appropriate precursor solution without acrylamide to a 15% arylamide solution. The gels were prepared in Petri dishes (60 mm × 15 mm) which were plasma oxidized prior to hydrogel formation. We prepared 4 mL of the 15% acrylamide solution in the Petri dish and started the hydrogel formation by the addition of 60 μL of a 10% ammonium persulfate (APS) solution in MQ water and 8 μL tetramethylethylenediamine (TEMED). The solution was mixed and heated for 10 minutes at 50 °C. The PAA hydrogel was cooled to room temperature for 30 min. We cut out hydrogel pieces with a scalpel, placed them in a Petri dish (35 mm × 10 mm) and sealed with Parafilm®. The gels were prepared freshly before each experiment.

### Spray deposition

Pulsed spray deposition of 200 μL of 3 M stock solutions of EDC in MQ water was performed using the air atomizing nozzle JAUCO D555000 (Spraying Systems Co.) The respective solutions were sprayed on 15% PAA hydrogels containing different concentrations of the precursor, which were placed in a Petri dish (35 mm × 10 mm) at ambient temperature. Oil-free nitrogen was used as a carrier gas with a constant pressure set to 0.5 bar. The air atomizing nozzle was mounted on a custom-built spray coater at a distance of roughly 13.5 cm to the PAA gels. A magnetic valve of the type MEBH-5/2-1/8-B (Festo SE & Co. KG) was connected to a microcontroller and controlled the pulsed spray deposition with a periodicity of 50 ms spraying time followed by 450 ms of waiting time. The spray cone provided by the nozzle covered a circular area with a diameter of roughly 2.5 cm. In order to obtain an improved homogeneous coverage of the PAA hydrogels, the air atomizing nozzle was moved periodically within 1 cm by the spray coater, which was controlled by a second microcontroller. The obtained samples were sealed in closed Petri dishes (35 mm × 10 mm) with Parafilm® and analyzed by a webcam set-up and subsequent grey value analysis.

### Preparation of drug delivery platform

The drug-delivery emulsion was prepared by emulsifying the anhydride (60 mM) in MES-buffer (pH 6, 200 mM) with 40 μL of the drug stock solution and sonication for 2 min with a Branson UltrasonicsTM SonifierTM SFX250 at 25% in an ice bath. These anhydride/drug emulsions were prepared freshly for each experiment. 250 μL of the emulsion was then mixed with 250 μL of a precursor stock (32–44 mM) and 500 μL of a 2% agar–agar stock in MES-buffer (pH 6, 200 mM) heated to 90 °C, was added subsequently. Then, 60 μL of this mixture was prepared on the bottom of a 96-well plate. The emulgel was cooled down to room temperature and 120 μL of an 8.0–11.0 mM precursor stock was added as supernatant. The cumulative drug release was measured in the supernatant by HPLC. All experiments were performed at 25 °C in triplicate.

### HPLC

We used analytical HPLC (ThermoFisher, Vanquish Duo UHPLC, HPLC1) with a Hypersil Gold 100 × 2.1 mm C18 column (3 μm pore size) to monitor the concentration profiles of each reactant of the chemical reaction network. 1.0 mL samples were prepared into a screw cap HPLC vial following the sample preparation protocol described above. Samples were injected directly without any further dilution from the HPLC vial. We injected 2.5 μL for the detection of the precursor and anhydride. For the detection of EDC, we injected 0.1 μL. We used a UV/vis detector at 220 nm for detection. A linear gradient of MQ water: ACN with 0.1% TFA was used to separate the compounds. The separation method was based on a linear gradient from 60 : 40 to 2 : 98 for the anhydride and 95 : 5 to 2 : 98 for EDC in 5 min followed by 1 min at 2 : 98. Afterwards, the gradient changed from 2 : 98 back to 60 : 40 or 95 : 5 in 0.2 min and the column was equilibrated for 3.8 min. The drug and acid releases from the emulgel were determined by analytical HPLC (ThermoFisher, Dionex Ultimate 3000, HPLC2) with a Hypersil Gold 250 × 4.8 mm C18 column (5 μm pore size) using a linear gradient of MQ water and ACN, each with 0.1% TFA. All compounds were detected with an UV/vis detector at 220 nm (precursor) and 330 nm (Nimesulide). We used a gradient of MQ water : ACN from 98 : 2 to 2 : 98 in 13 min for the separation. We performed calibration curves of all the compounds in triplicates. Calibration values and retention times are given in ESI, Table S1.†

### Nuclear resonance spectroscopy (NMR)

We recorded NMR spectra on a Bruker AVIII-400 at 25 °C and a frequency of 400 MHz. Chemical shifts *δ* are reported in ppm and are referred to the residual solvent peak of the used deuterated solvent (chloroform-d_1_ 7.26 ppm for ^1^H-NMR). We abbreviated the signal multiplets as followed: s-singulet, d-dublet, t-triplet, m-multiplet. The coupling constant *J* is stated as average value in Hz and refers to coupling between two protons.

^1^H NMR (anhydride) 400 MHz, CDCl_3_):*δ* (ppm) = 5.61 (dt, ^3^*J*_H–H_ = 13.8, 6.6 Hz, 1H; CH=C), 5.29 (dt, ^3^*J*_H–H_ = 15.3, 7.6 Hz, 1H; CH=C), 3.20 (m, 1H; CH), 3.01 (dd, ^2,3^*J*_H–H_ = 18.9, 9.7 Hz, 1H; CH_2_), 2.73 (dd, ^2,3^*J*_H–H_ = 18.7, 5.9 Hz, 1H; CH_2_), 2.49 (m, 2H, CH_2_), 2.01 (dt, ^3^*J*_H–H_ = 7.2, 6.9 Hz, 2H; CH_2_), 1.27 (m, 10H; (CH_2_)_4_), 0.88 (t, ^3^*J*_H–H_ = 6.6 Hz, 3H; CH_3_) (see ESI, Fig. 13†).

^1^H NMR (precursor) 400 MHz, CDCl_3_):*δ* (ppm) = 5.51 (dt, ^3^*J*_H–H_ = 13.7, 6.8 Hz, 1H; CH=C), 5.31 (dt, ^3^*J*_H–H_ = 14.9, 7.0 Hz, 1H; CH=C), 2.89 (m, 1H; CH), 2.67 (dd, ^2,3^*J*_H–H_ = 17.5, 10.7 Hz, 1H; CH_2_), 2.53 (dd, ^2,3^*J*_H–H_ = 17.3, 3.9 Hz, 1H; CH_2_), 2.44 (m, 1H, CH_2_), 2.22 (m, 1H; CH_2_), 1.99 (dt, ^3^*J*_H–H_ = 7.0 Hz, 2H; CH_2_), 1.27 (m, 10H; (CH_2_)_4_), 0.88 (t, ^3^*J*_H–H_ = 6.6 Hz, 3H; CH_3_) (see ESI, Fig. 14†).

### UV/vis-spectroscopy

A Multiskan FC microplate reader (ThermoFisher) was used for UV/vis measurements. For the sample preparation a 96-well-plate (tissue culture plate non-treated) was used. Measurements were performed in triplicates at a wavelength of 600 nm and 25 °C.

## Data availability

The authors confirm that the data supporting the findings of this study are available within the article and its ESI.

## Author contributions

P. S. S., J. B. and P. M.-B. designed the experiments. P. S. S., L. T. and J. E. H performed the experiments. P. S. S. and J. B. wrote the manuscript. All authors have given approval to the final version of the manuscript.

## Conflicts of interest

There are no conflicts to declare.

## Supplementary Material

SC-012-D1SC02561A-s001
