# Supplementary material for: Chemically fueled materials with a self-immolative mechanism: transient materials with a fast on/off response
Source: Chem Sci. 2021 Jun 21;12(29):9969–76. doi: 10.1039/d1sc02561a (PMC8317627; doi:10.1039/d1sc02561a)
Supplement: SC-012-D1SC02561A-s001 [file SC-012-D1SC02561A-s001.pdf]

Electronic Supplementary Material (ESI) for Chemical Science.
This journal is © The Royal Society of Chemistry 2021

Supporting Information

Chemically Fueled Materials with a Self-Immolative Mechanism: Transient Materials with a Fast On/Off Response

*Patrick S. Schwarz, Laura Tebcharani, Julian E. Heger, Prof. Peter Müller-Buschbaum, Prof. Job Boekhoven**

P. S. Schwarz, L. Tebcharani, Prof. J. Boekhoven
Department of Chemistry, Technical University of Munich, Lichtenbergstraße 4, 85748 Garching, Germany.

Prof. J. Boekhoven
Institute for Advanced Study, Technical University of Munich, Lichtenbergstraße 2a, 85748 Garching, Germany.
E-mail: job.boekhoven@tum.de

J. E. Heger, Prof. P. Müller-Buschbaum
Department of Physics, Technical University of Munich, James-Franck-Straße 1, 85748 Garching, Germany.

Prof. P. Müller-Buschbaum
Heinz Maier-Leibnitz Zentrum (MLZ), Technical University of Munich, Lichtenbergstraße 1, 85748 Garching, Germany

1. Supporting Tables

Supporting Table 1: Characterization of compounds.

Name	Structure	Mass calc. [g/mol]	Mass obs. [g/mol]	Retention time [min]	Calibration value [mAU mM^{-1}]
**Precursor**	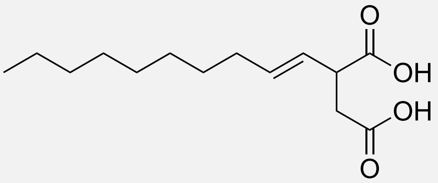	256.17<br>$C_{14}H_{24}O_4$	257.1<br>$[M+H]^+$	5.22<br>(HPLC1)<br><br>11.79<br>(HPLC2)	0.76<br>(HPLC1)<br><br>3.25<br>(HPLC2)
**Anhydride**	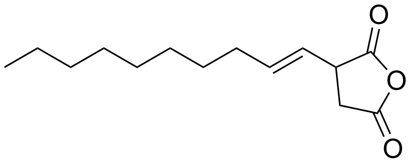	238.16<br>$C_{14}H_{22}O_3$	239.1<br>$[M+H]^+$	6.71<br>(HPLC1)	1.77<br>(HPLC1)
**Nimesulide**	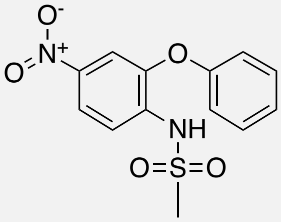	308.05<br>$C_{13}H_{12}N_2O_5S$	309.1<br>$[M+H]^+$	10.99<br>(HPLC2)	0.15<br>(HPLC2)

Supporting Table 2: Rate constants used in the kinetic model.

c_0(EDC) [mM]	c(Precursor) [mM]	k_1 [M^{-1} s^{-1}]	k_2	k_3	k_4 [s^{-1}]	CMC [mM]	S_0 [mM]	SC
**5**	20	0.5	$1*k_1$	$0.3*k_1$	3.5E-3	16.5	1.10E-1	0.1
**6**	20	0.5	$1*k_1$	$0.3*k_1$	3.5E-3	16.5	0.90E-1	0.1
**7**	20	0.5	$1*k_1$	$0.3*k_1$	3.5E-3	16.5	0.65E-1	0.1
**8**	7.5	0.5	$1*k_1$	$0.3*k_1$	3.5E-3	16.5	0.25E-1	0.1
**8**	20	0.5	$1*k_1$	$0.3*k_1$	3.5E-3	16.5	0.55E-1	0.1
**9**	20	0.5	$1*k_1$	$0.3*k_1$	3.5E-3	16.5	0.53E-1	0.1

2. Supporting Figures

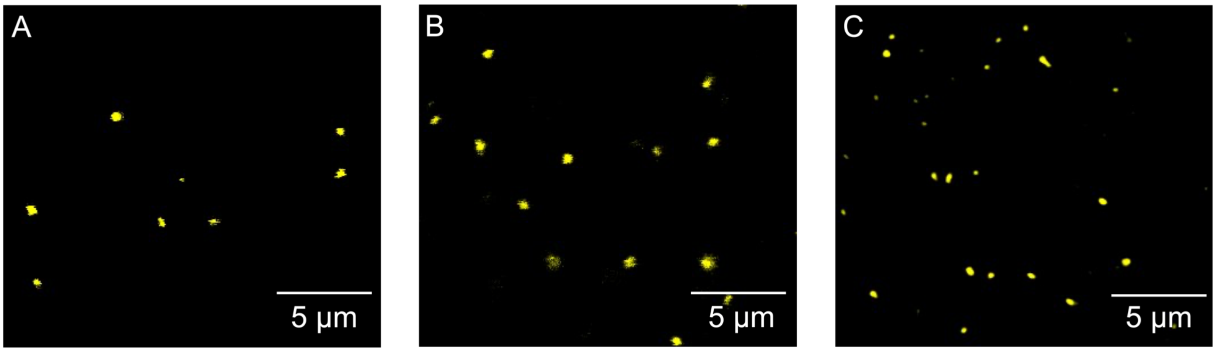

Supporting Figure 1: Confocal micrographs of **A**) 7.5 mM precursor and 2 mM EDC, **B**) 7.5 mM precursor and 8 mM EDC and **C**) 20 mM precursor and 8 mM EDC.

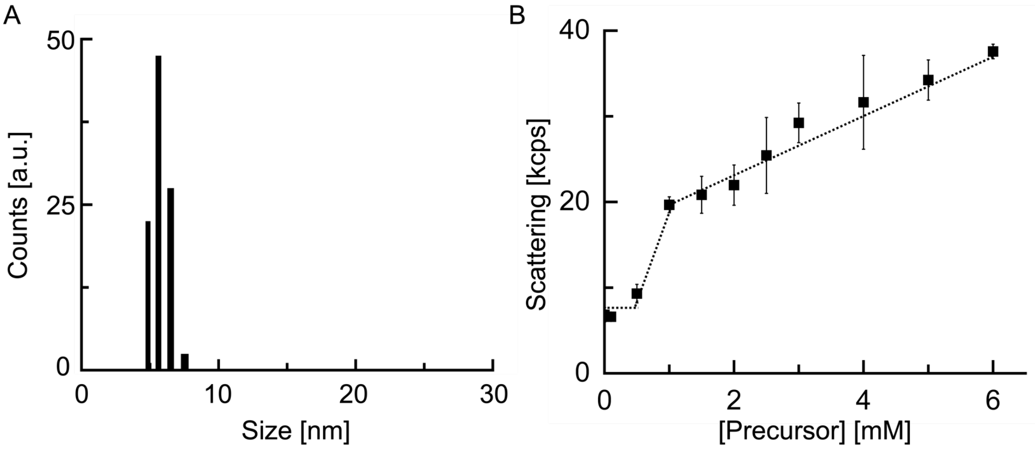

Supporting Figure 2: **A**) Size distribution of the hydrodynamic diameter of 20 mM precursor in 200 mM MES. **B**) Scattering for different amounts of precursor. Lines are added to guide the eye.

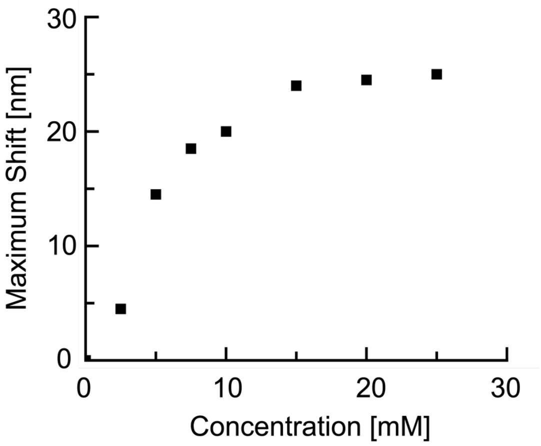

Supporting Figure 3: Nile Red fluorescence assay showing a fluorescence maximum shift for different precursor concentrations characteristic for incorporation into micelles.

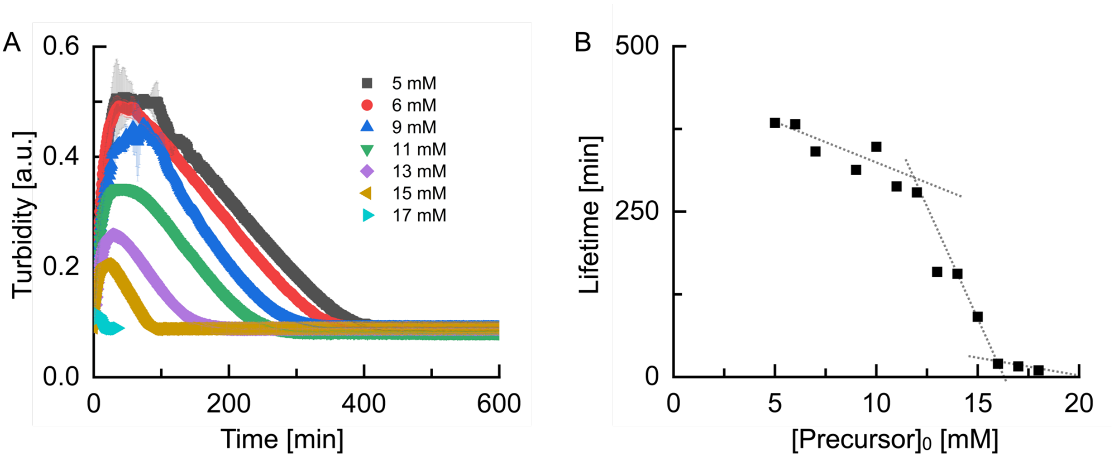

Supporting Figure 4: **A**) Turbidity at 600 nm for different precursor concentrations fueled with 2 mM EDC. **B**) Lifetime of the turbidity as a function of initial precursor concentration. Lines are added to guide the eye.

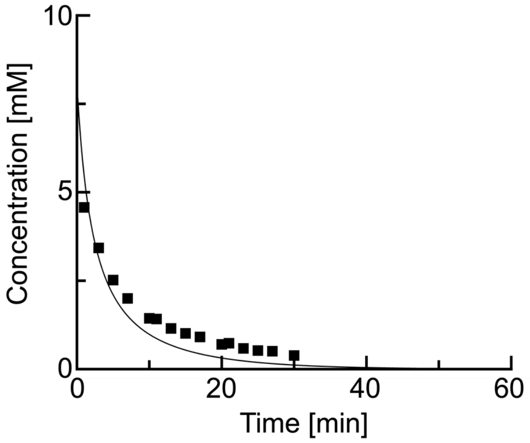

Supporting Figure 5: EDC consumption of 7.5 mM precursor fueled with 8 mM EDC. Markers represent HPLC data; solid lines represent data calculated by the kinetic model.

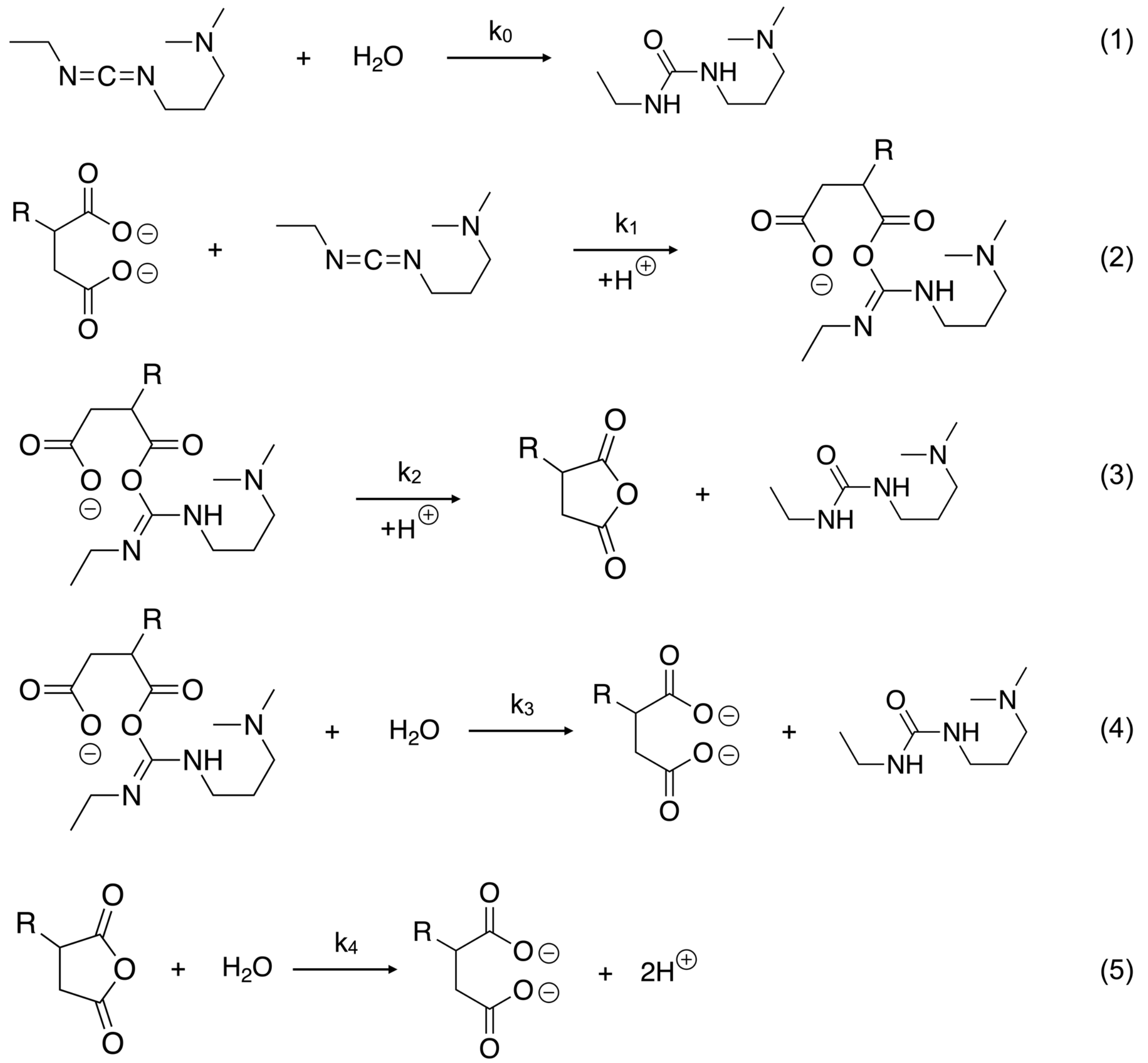

Supporting Figure 6: **Chemical reactions considered in the kinetic model**. Reaction (1) shows the direct hydrolysis of EDC. Reaction (2) shows the activation reaction of the succinate precursor with EDC. Reaction (3) shows the intramolecular anhydride formation reaction. Reaction (4) shows the direct hydrolysis of *O*-acylisourea. Reaction (5) shows the hydrolysis of the anhydride.

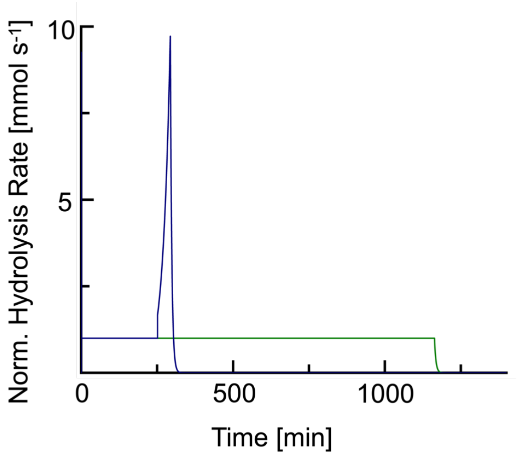

Supporting Figure 7: Normalized hydrolysis rate constant of 20 mM precursor (blue) and 7.5 mM precursor fueled with 8 mM EDC.

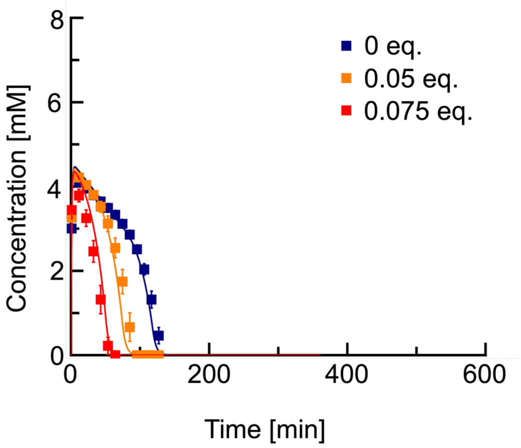

Supporting Figure 8: Anhydride concentration profiles of 20 mM precursor fueled with 6 mM EDC and seeded with different amounts of precursor. Markers represent HPLC data; solid lines represent data calculated by the kinetic model.

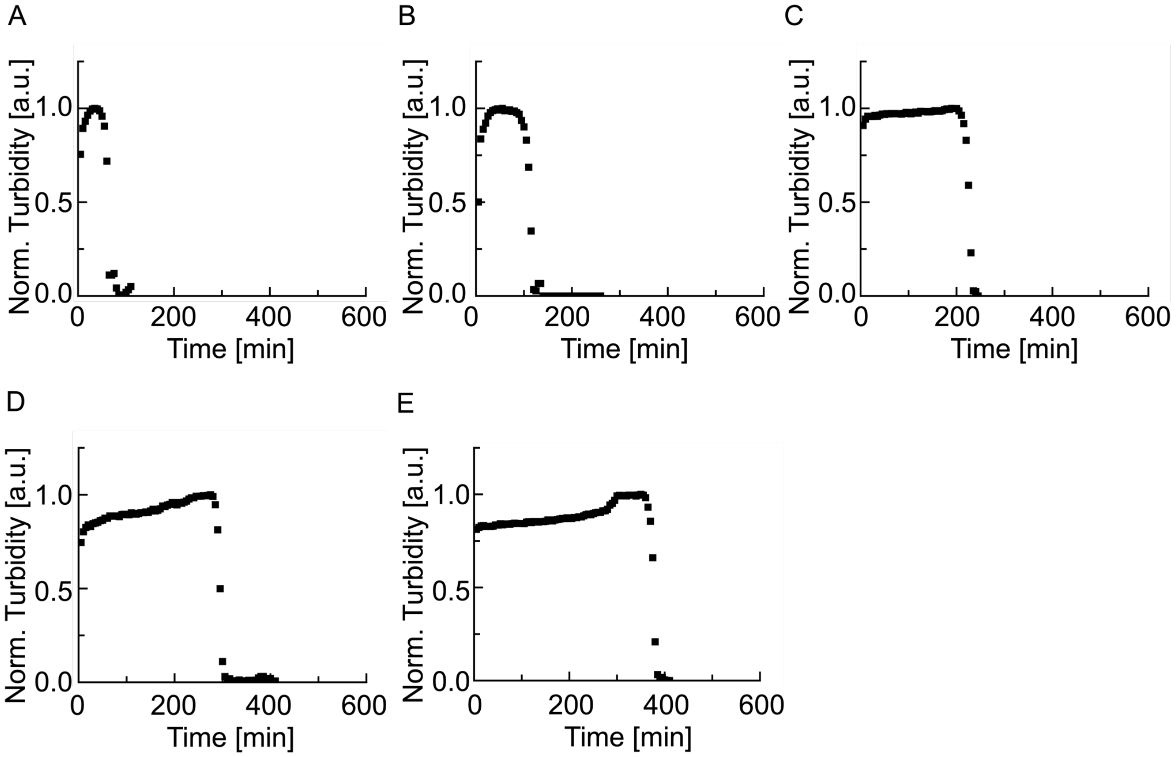

Supporting Figure 9: Normalized turbidity of 20 mM precursor fueled with **A**) 5 mM, **B**) 6 mM, **C**) 7 mM, **D**) 8 mM and **E**) 9 mM EDC.

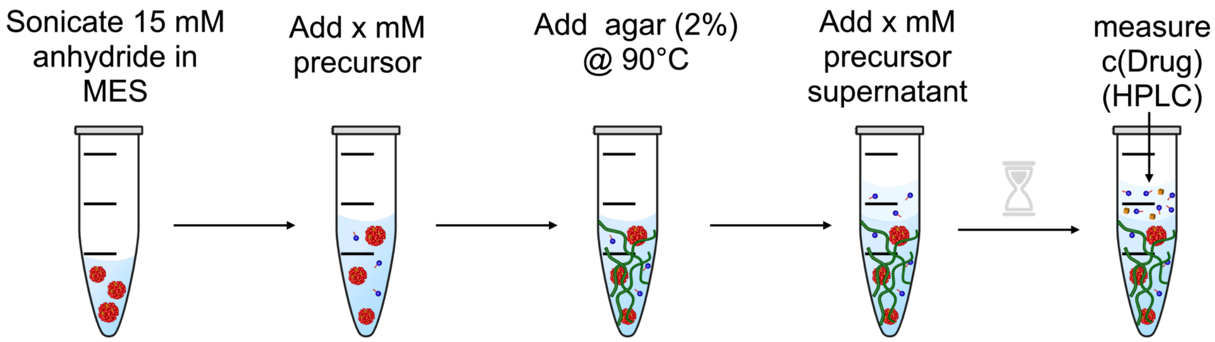

Supporting Figure 10: Schematic representation of the preparation of a self-immolative drug delivery platform.

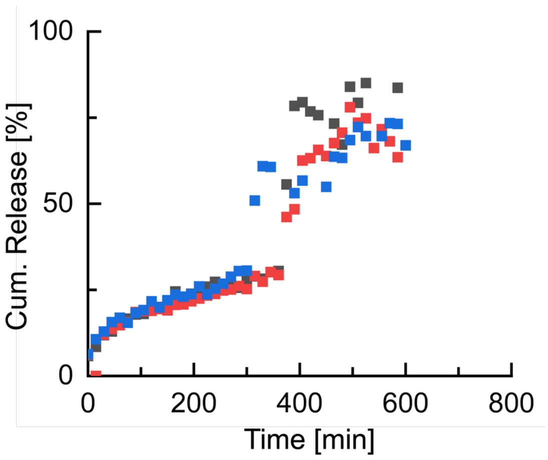

Supporting Figure 11: Triplicate measurements of the cumulative drug release of an agar agar hydrogel with 25 µM Nimesulide in an emulsion of 15 mM anhydride and 9 mM precursor

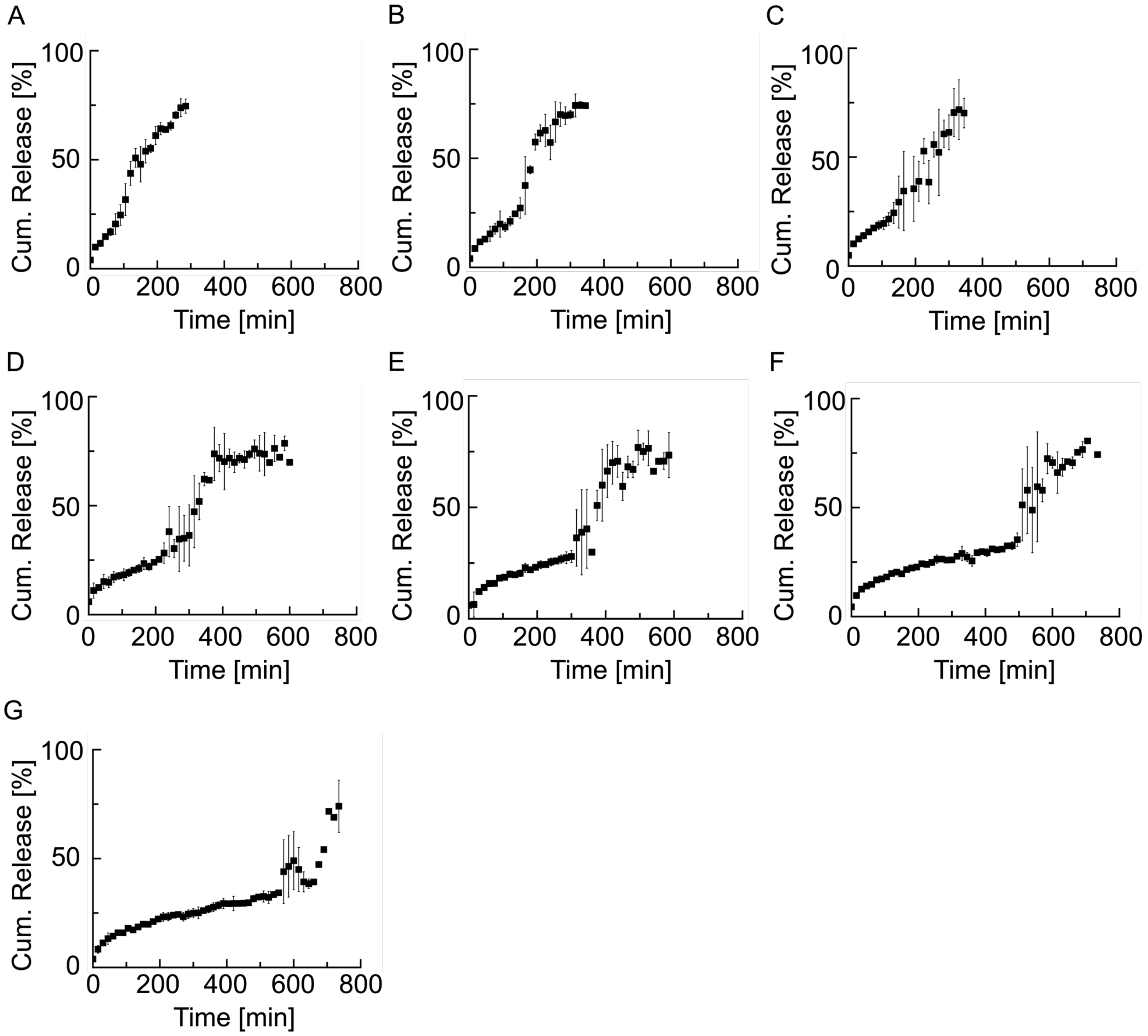

Supporting Figure 12: Cumulative drug release of an agar agar hydrogel with 25 µM Nimesulide in an emulsion of 15 mM anhydride and **A**) 11 mM precursor, **B**) 10.5 mM precursor, **C**) 10 mM precursor, **D**) 9.5 mM precursor, **E**) 9 mM precursor, **F**) 8.5 mM precursor and **G**) 8 mM precursor.

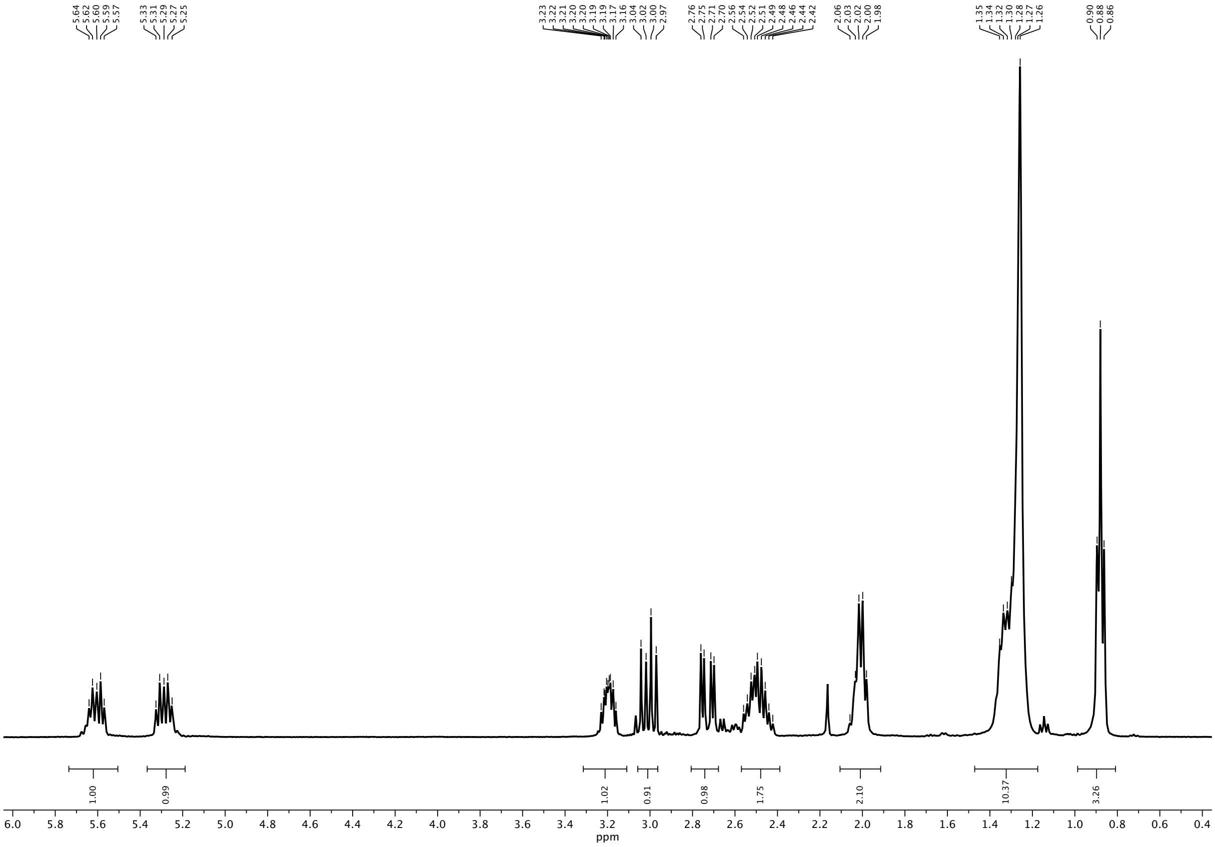

Supporting Figure 13: ^{1}H-NMR ($CDCl_3$, 400 MHz) spectrum of the anhydride.

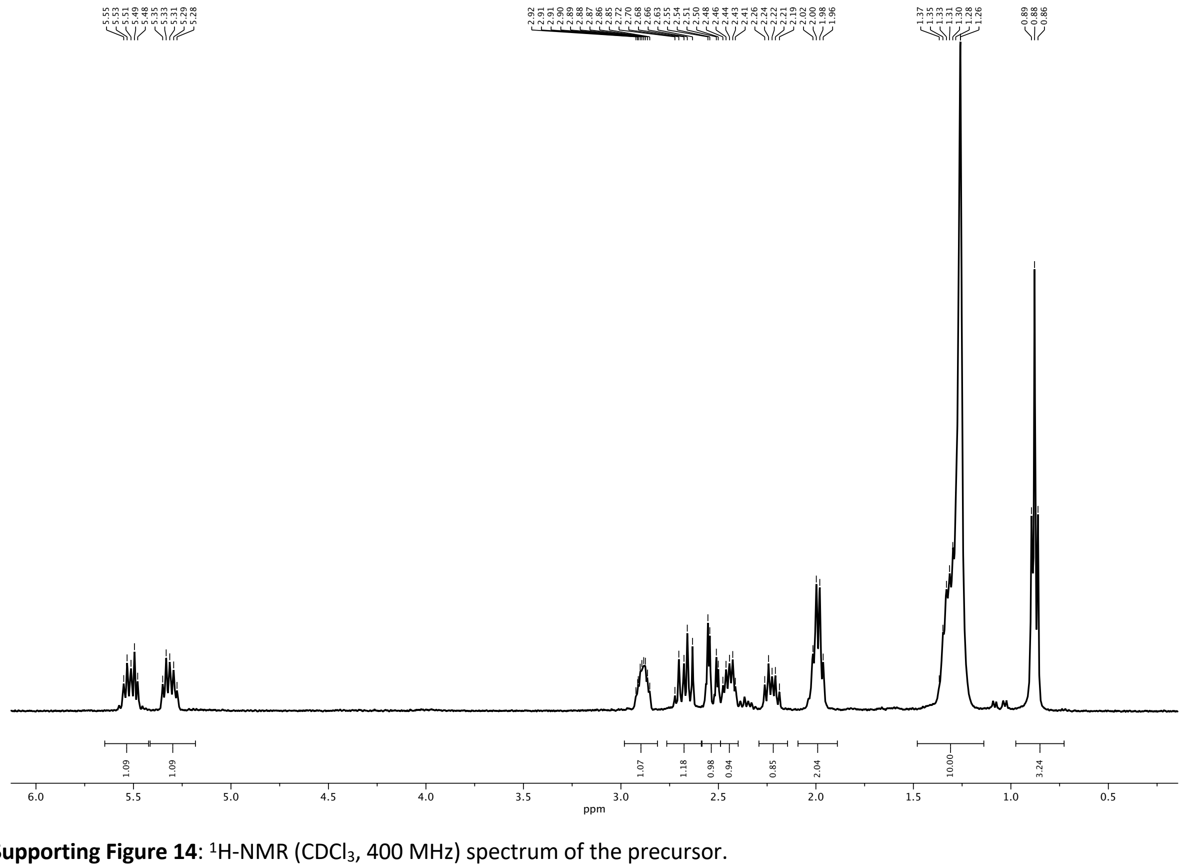

Supporting Figure 14: ^{1}H-NMR ($CDCl_3$, 400 MHz) spectrum of the precursor.

Supporting Notes

Critical Micelle Concentration in an Emulsion. The critical micelle concentration (*CMC*) of a surfactant can be determined by various techniques, such as surface tension, conductivity, dynamic light scattering (DLS) and dye solubilization spectrophotometry. However, the accurate determination of the *CMC* of a surfactant in the presence of oil droplets remains challenging. Standard techniques like dynamic light scattering and photon correlation spectroscopy fail to analyze oil and micelles simultaneously. Moreover, oil droplets have an influence on the surface tension, conductivity and solubilize hydrophobic dyes which prevents other analytical techniques. We considered the lifetime of the emulsion as an indirect measurement of the formation of micelles when we fueled different amounts of precursor with 2 mM EDC (see Supporting Figure 4). We found that the lifetime decreased linearly when we increased the precursor concentration from 5 mM to 12 mM which we attributed to a salting-in effect of the additional precursor which slightly increases the solubility of the anhydride. However, at precursor concentrations higher than 12 mM, we observed a significant decrease of the lifetime of the turbidity by roughly 50 % which indicated the formation of micelles. In conclusion, when fueled with 2 mM EDC and subtracting an anhydride yield of roughly 1.5 mM, the *CMC* of the precursor is roughly 11 mM.

Kinetic Model. The concentration of each reactant (anhydride, precursor, *O*-acylisourea and EDC) was described for every second in the reaction cycle in a kinetic *Matlab* model. For the description of the chemical reaction network, a basis of five differential equations for the five underlying chemical reactions was used for the calculations of concentrations (see Supporting Figure 5): the direct hydrolysis of EDC r_0 (1), the activation of the precursor r_1 to form the intermediate product O-acylisourea (2), the anhydride formation r_2 (3), the direct hydrolysis of the intermediate product *O*-acylisourea r_3 (4), the hydrolysis of the anhydride r_4 (7,8). For the implementation of the self-amplifying decay, we defined the effective solubility of the anhydride *COOOC(i)* as a variable $S_{eff}(i)$ which is constant below the *CMC* of the precursor *COOH(i)* (5). Above the *CMC*, the solubility of the anhydride changes as a function of precursor concentration *COOH(i)* (6). We defined a solubilization capacity (*SC*, see Supporting Table S3) which can be seen as the effectiveness of the surfactant precursor which increases the solubility of the anhydride in dependence on the number of precursor molecules above the *CMC*. In other words, the solubilization factor of 0.1 means that 10 molecules of precursor are necessary to dissolve 1 molecule of anhydride. We used two differential equations for the hydrolysis rate of the anhydride above and below its effective solubility $S_{eff}(i)$. Above the solubility, the hydrolysis rate is constant as long as $S_{eff}(i)$ is constant (see 5 and 6) (7). Below the solubility, the hydrolysis rate becomes a first order rate which is dependent on the anhydride concentration *COOC(i)* (8).

$$r_0(i) = k_0 \cdot EDC(i) \tag{1}$$

$$r_1(i) = k_1 \cdot EDC(i) \cdot COOH(i) \tag{2}$$

$$r_2(i) = k_2 \cdot COOEDC(i) \tag{3}$$

$$r_3(i) = k_3 \cdot COOEDC(i) \tag{4}$$

$$S_{eff}(i) = S_0, \qquad if\ COOH(i) < CMC \tag{5}$$

$$S_{eff}(i) = S_0 + \big(SC * (COOH(i) - CMC)\big), \qquad if\ COOH(i) > CMC \tag{6}$$

$$r_4(i) = k_4 \cdot S_{eff}(i), \qquad if\ COOOC(i) > S_{eff}(i) \tag{7}$$

$$r_4(i) = k_4 \cdot COOOC(i), \qquad if\ COOOC(i) < S_{eff}(i) \tag{8}$$
